# Supplementary material for: Palatal morphology predicts the paleobiology of early salamanders
Source: eLife. 2022 May 16;11:e76864. doi: 10.7554/eLife.76864 (PMC9170251; doi:10.7554/eLife.76864)
Supplement: Supplementary file 1. — (A) Scores of the principal components generated from the standard principal component analysis (PCA) on the 24-landmark-dataset across 70 specimens, and the phylomorphospace analysis (PA), the phylogenetically aligned component analysis (PaCA), and the phylogenetic principal component analysis (Phylo-PCA) across 34 species. Abbreviations: Cumu. RV, Cumulative RV; Cumu. P., Cumulative Proportion; Eigenva., Eigenvalue; Prop. Co., Proportion of Covariance; Prop. V., Proportion of Variance; RV by Co., RV by Component; Sing. V., Singular Value. (B) Pairwise comparison and corresponding p-values of morphological disparity of the palate calculated as Procrustes variances for 34 species grouped by ecological preference, life history strategy and taxonomic affiliation. *, p-value ≤ 0.05. Abbreviations: cryptobran., cryptobranchoids. (C) Pairwise comparison and corresponding p-values of single landmark point of the palate calculated as Procrustes variances (×E-4) for 34 species grouped by ecological preference. *, p≤0.05; **, p≤0.01; ***, p≤0.001. (D) Pairwise comparison and corresponding p-values of single landmark point of the palate calculated as Procrustes variances (×E-5) for 34 species grouped by life history strategy. *, p≤0.05. (E) Pairwise comparison and corresponding p-values of single landmark point of the palate calculated as Procrustes variances (×E-5) for 34 species grouped by taxonomic affiliation. *, p≤0.05; **, p≤0.01. (F) Absolute morphological disparity of the palate calculated as Procrustes variances for 34 species grouped by ecology, life history strategy and taxonomic affiliations. (G) Absolute morphological disparity calculated as Procrustes variances and evolutionary rates for each landmark point of the palate across 34 species grouped by ecology, life history and taxonomic affiliations. (H) Comparison of evolutionary rates of the palate and centroid size for 34 species grouped by ecology, life history and taxonomic affiliations. *, p≤0.05; **, p≤0 [file elife-76864-supp1.docx]

Supplementary file 1 for

**Palatal morphology predicts the palaeobiology of early salamanders**

**Jia Jia, Guangzhao Li, and Ke-Qin Gao**

This PDF file contains supplementary file 1A to 1K.

A Scores of the principle components generated from the standard principle component analysis (PCA) on the 24-landmark-dataset across 70 specimens, and the phylomorphospace analysis (PA), the phylogenetically aligned component analysis (PaCA), and the phylogenetic principal component analysis (Phylo-PCA) across 34 species. Abbreviations: Cumu. RV, Cumulative RV; Cumu. P., Cumulative Proportion; Eigenva., Eigenvalue; Prop. Co., Proportion of Covariance; Prop. V., Proportion of Variance; RV by Co., RV by Component; Sing. V., Singular Value.

|  | Standard PCA of 70 specimens | | |  | Phylomorphospace analysis across 34 species | | | |
| --- | --- | --- | --- | --- | --- | --- | --- | --- |
| PCs | Eigenva. | Prop. V. | Cumu. P. | **PCs** | Eigenva. | Prop. V. | Cumulative Proportion | |
| Comp1 | 6.73E-03 | 3.48E-01 | 3.48E-01 | **Comp1** | 7.63E-03 | 3.75E-01 | 3.75E-01 | |
| Comp2 | 4.16E-03 | 2.15E-01 | 5.62E-01 | **Comp2** | 4.35E-03 | 2.13E-01 | 5.88E-01 | |
| Comp3 | 2.32E-03 | 1.20E-01 | 6.82E-01 | **Comp3** | 2.38E-03 | 1.17E-01 | 7.05E-01 | |
| Comp4 | 1.61E-03 | 8.32E-02 | 7.65E-01 | **Comp4** | 1.75E-03 | 8.57E-02 | 7.91E-01 | |
| Comp5 | 9.94E-04 | 5.13E-02 | 8.17E-01 | **Comp5** | 1.05E-03 | 5.17E-02 | 8.43E-01 | |
| Comp6 | 8.07E-04 | 4.17E-02 | 8.58E-01 | **Comp6** | 8.12E-04 | 3.99E-02 | 8.83E-01 | |
| Comp7 | 5.90E-04 | 3.05E-02 | 8.89E-01 | **Comp7** | 5.97E-04 | 2.93E-02 | 9.12E-01 | |
| Comp8 | 4.44E-04 | 2.29E-02 | 9.12E-01 | **Comp8** | 5.09E-04 | 2.50E-02 | 9.37E-01 | |
| Comp9 | 3.95E-04 | 2.04E-02 | 9.32E-01 | **Comp9** | 2.81E-04 | 1.38E-02 | 9.51E-01 | |
| Comp10 | 2.59E-04 | 1.34E-02 | 9.45E-01 | **Comp10** | 2.12E-04 | 1.04E-02 | 9.61E-01 | |
| Comp11 | 2.08E-04 | 1.07E-02 | 9.56E-01 | **Comp11** | 1.81E-04 | 8.90E-03 | 9.70E-01 | |
| Comp12 | 1.95E-04 | 1.01E-02 | 9.66E-01 | **Comp12** | 1.44E-04 | 7.05E-03 | 9.77E-01 | |
| Comp13 | 1.34E-04 | 6.93E-03 | 9.73E-01 | **Comp13** | 1.22E-04 | 6.01E-03 | 9.83E-01 | |
| Comp14 | 1.29E-04 | 6.66E-03 | 9.80E-01 | **Comp14** | 1.01E-04 | 4.95E-03 | 9.88E-01 | |
| Comp15 | 9.31E-05 | 4.80E-03 | 9.85E-01 | **Comp15** | 7.72E-05 | 3.79E-03 | 9.92E-01 | |
| Comp16 | 7.79E-05 | 4.02E-03 | 9.89E-01 | **Comp16** | 5.60E-05 | 2.75E-03 | 9.95E-01 | |
| Comp17 | 7.21E-05 | 3.72E-03 | 9.92E-01 | **Comp17** | 3.55E-05 | 1.75E-03 | 9.96E-01 | |
| Comp18 | 5.82E-05 | 3.00E-03 | 9.95E-01 | **Comp18** | 3.07E-05 | 1.51E-03 | 9.98E-01 | |
| Comp19 | 3.81E-05 | 1.97E-03 | 9.97E-01 | **Comp19** | 1.96E-05 | 9.62E-04 | 9.99E-01 | |
| Comp20 | 2.35E-05 | 1.21E-03 | 9.98E-01 | **Comp20** | 1.17E-05 | 5.76E-04 | 9.99E-01 | |
| Comp21 | 1.87E-05 | 9.67E-04 | 9.99E-01 | **Comp21** | 7.08E-06 | 3.48E-04 | 1.00E+00 | |
| Comp22 | 1.10E-05 | 5.67E-04 | 1.00E+00 | **Comp22** | 4.71E-06 | 2.32E-04 | 1.00E+00 | |
| Comp23 | 2.16E-18 | 1.12E-16 | 1.00E+00 |  |  |  |  |  |
|  | **PaCA analysis across 34 species** | | | | | **Phylo-PCA across 34 species** | | |
| PCs | Sing. V. | Prop. Co. | Cumu. P. | RV by Co. | Cumu. RV | Eigenva. | Prop. V. | Cumu. P. |
| Comp1 | 1.72E-02 | 8.43E-01 | 8.43E-01 | 3.55E-02 | 3.55E-02 | 1.68E-04 | 3.94E-01 | 3.94E-01 |
| Comp2 | 2.96E-03 | 1.45E-01 | 9.88E-01 | 6.13E-03 | 4.17E-02 | 7.84E-05 | 1.84E-01 | 5.79E-01 |
| Comp3 | 9.69E-05 | 4.76E-03 | 9.93E-01 | 2.01E-04 | 4.19E-02 | 3.81E-05 | 8.95E-02 | 6.68E-01 |
| Comp4 | 6.80E-05 | 3.34E-03 | 9.96E-01 | 1.41E-04 | 4.20E-02 | 3.22E-05 | 7.57E-02 | 7.44E-01 |
| Comp5 | 3.99E-05 | 1.96E-03 | 9.98E-01 | 8.27E-05 | 4.21E-02 | 2.69E-05 | 6.34E-02 | 8.07E-01 |
| Comp6 | 1.83E-05 | 8.98E-04 | 9.99E-01 | 3.79E-05 | 4.21E-02 | 1.77E-05 | 4.16E-02 | 8.49E-01 |
| Comp7 | 6.67E-06 | 3.28E-04 | 9.99E-01 | 1.38E-05 | 4.21E-02 | 1.51E-05 | 3.55E-02 | 8.84E-01 |
| Comp8 | 4.14E-06 | 2.03E-04 | 9.99E-01 | 8.57E-06 | 4.21E-02 | 1.12E-05 | 2.64E-02 | 9.11E-01 |
| Comp9 | 3.45E-06 | 1.70E-04 | 1.00E+00 | 7.15E-06 | 4.22E-02 | 8.46E-06 | 1.99E-02 | 9.31E-01 |
| Comp10 | 2.19E-06 | 1.08E-04 | 1.00E+00 | 4.55E-06 | 4.22E-02 | 6.60E-06 | 1.55E-02 | 9.46E-01 |
| Comp11 | 1.80E-06 | 8.83E-05 | 1.00E+00 | 3.72E-06 | 4.22E-02 | 5.36E-06 | 1.26E-02 | 9.59E-01 |
| Comp12 | 1.43E-06 | 7.02E-05 | 1.00E+00 | 2.96E-06 | 4.22E-02 | 3.66E-06 | 8.61E-03 | 9.67E-01 |
| Comp13 | 7.05E-07 | 3.46E-05 | 1.00E+00 | 1.46E-06 | 4.22E-02 | 3.35E-06 | 7.87E-03 | 9.75E-01 |
| Comp14 | 6.79E-07 | 3.33E-05 | 1.00E+00 | 1.41E-06 | 4.22E-02 | 2.95E-06 | 6.94E-03 | 9.82E-01 |
| Comp15 | 2.19E-07 | 1.08E-05 | 1.00E+00 | 4.54E-07 | 4.22E-02 | 2.67E-06 | 6.27E-03 | 9.88E-01 |
| Comp16 | 1.64E-07 | 8.05E-06 | 1.00E+00 | 3.39E-07 | 4.22E-02 | 1.34E-06 | 3.16E-03 | 9.92E-01 |
| Comp17 | 1.25E-07 | 6.15E-06 | 1.00E+00 | 2.59E-07 | 4.22E-02 | 1.21E-06 | 2.83E-03 | 9.94E-01 |
| Comp18 | 7.07E-08 | 3.47E-06 | 1.00E+00 | 1.46E-07 | 4.22E-02 | 9.86E-07 | 2.32E-03 | 9.97E-01 |
| Comp19 | 6.89E-08 | 3.38E-06 | 1.00E+00 | 1.43E-07 | 4.22E-02 | 6.69E-07 | 1.57E-03 | 9.98E-01 |
| Comp20 | 2.04E-08 | 1.00E-06 | 1.00E+00 | 4.22E-08 | 4.22E-02 | 3.71E-07 | 8.71E-04 | 9.99E-01 |
| Comp21 | 1.64E-08 | 8.05E-07 | 1.00E+00 | 3.39E-08 | 4.22E-02 | 2.73E-07 | 6.43E-04 | 1.00E+00 |
| Comp22 | 7.56E-09 | 3.71E-07 | 1.00E+00 | 1.57E-08 | 4.22E-02 | 1.01E-07 | 2.38E-04 | 1.00E+00 |

B Pairwise comparison and corresponding *p*-values of morphological disparity of the palate calculated as procrustes variances for 34 species grouped by ecological preference, life history strategy and taxonomic affiliation. *, *p* value <= 0.05. Abbreviations: cryptobran., cryptobranchoids.

| whole palate | Aquatic | Semiaquatic | Terrestrial | whole palate | Metamorphosis | Neoteny |
| --- | --- | --- | --- | --- | --- | --- |
| Aquatic | 0 |  |  | Metamorphosis | 0 |  |
| Semiaquatic | 4.26E-03 | 0 |  | Neoteny | 2.40E-03 | 0 |
| Terrestrial | 5.71E-03 | 1.45E-03 | 0 |  |  |  |
| vomer | Aquatic | Semiaquatic | Terrestrial | vomer | Metamorphosis | Neoteny |
| Aquatic | 0 |  |  | Metamorphosis | 0 |  |
| Semiaquatic | 1.27E-03 | 0 |  | Neoteny | 1.16E-03 | 0 |
| Terrestrial | 2.70E-03 | 1.43E-03 | 0 |  |  |  |
| parasphenoid | Aquatic | Semiaquatic | Terrestrial | parasphenoid | Metamorphosis | Neoteny |
| Aquatic | 0 |  |  | Metamorphosis | 0 |  |
| Semiaquatic | 1.71E-03 | 0 |  | Neoteny | 6.88E-05 | 0 |
| Terrestrial | 3.10E-04 | 1.41E-03 | 0 |  |  |  |
| whole palate | basal cryptobran. | Pancryptobrancha | Hynobiidae | non-cryptobran. | Stem hynobiids | |
| basal cryptobran. | 0 |  |  |  |  | |
| Pancryptobrancha | 1.40E-03 | 0 |  |  |  | |
| Hynobiidae | 4.23E-03 | 5.63E-03 | 0 |  |  | |
| non-cryptobran. | 6.51E-03 | 7.91E-03 | 2.28E-03 | 0 |  | |
| Stem hynobiids | 4.65E-03 | 6.05E-03 | 4.18E-04 | 1.86E-03 | 0 | |
| vomer | basal cryptobran. | Pancryptobrancha | Hynobiidae | non-cryptobran. | Stem hynobiids | |
| basal cryptobran. | 0 |  |  |  |  | |
| Pancryptobrancha | 4.26E-05 | 0 |  |  |  | |
| Hynobiidae | 2.36E-03 | 2.40E-03 | 0 |  |  | |
| non-cryptobran. | 2.98E-03 | 3.02E-03 | 6.19E-04 | 0 |  | |
| Stem hynobiids | 1.87E-03 | 1.91E-03 | 4.90E-04 | 1.11E-03 | 0 | |
| parasphenoid | basal cryptobran. | Pancryptobrancha | Hynobiidae | non-cryptobran. | Stem hynobiids | |
| basal cryptobran. | 0 |  |  |  |  | |
| Pancryptobrancha | 1.31E-03 | 0 |  |  |  | |
| Hynobiidae | 4.84E-04 | 8.27E-04 | 0 |  |  | |
| non-cryptobran. | 5.54E-04 | 1.87E-03 | 1.04E-03 | 0 |  | |
| Stem hynobiids | 9.15E-04 | 2.23E-03***** | 1.40E-03***** | 3.61E-04 | 0 | |

C Pairwise comparison and corresponding *p*-values of single landmark point of the palate calculated as procrustes variances (× E-4) for 34 species grouped by ecological preference. *, *p* <= 0.05; **, *p* <= 0.01; ***, *p* <= 0.001.

| LM 1, 14 | Aquatic | Semiaquatic | Terrestrial | LM 2, 13 | Aquatic | Semiaquatic | Terrestrial |
| --- | --- | --- | --- | --- | --- | --- | --- |
| Aquatic | 0 |  |  | Aquatic | 0 |  |  |
| Semiaquatic | 8.03**^***^** | 0 |  | Semiaquatic | 0.88 | 0 |  |
| Terrestrial | 0.48 | 7.55^***^ | 0 | Terrestrial | 0.26 | 1.14 | 0 |
| LM 3, 12 | Aquatic | Semiaquatic | Terrestrial | LM 4, 11 | Aquatic | Semiaquatic | Terrestrial |
| Aquatic | 0 |  |  | Aquatic | 0 |  |  |
| Semiaquatic | 1.23 | 0 |  | Semiaquatic | 2.41 | 0 |  |
| Terrestrial | 0.02 | 1.24 | 0 | Terrestrial | 0.93 | 1.48 | 0 |
| LM 5, 10 | Aquatic | Semiaquatic | Terrestrial | LM 6, 9 | Aquatic | Semiaquatic | Terrestrial |
| Aquatic | 0 |  |  | Aquatic | 0 |  |  |
| Semiaquatic | 3.10 | 0 |  | Semiaquatic | 8.80 | 0 |  |
| Terrestrial | 16.29 | 19.39 | 0 | Terrestrial | 10.62^**^ | 1.81 | 0 |
| LM 7, 8 | Aquatic | Semiaquatic | Terrestrial | LM 15 | Aquatic | Semiaquatic | Terrestrial |
| Aquatic | 0 |  |  | Aquatic | 0 |  |  |
| Semiaquatic | 3.06 | 0 |  | Semiaquatic | 6.53^**^ | 0 |  |
| Terrestrial | 1.04 | 2.01 | 0 | Terrestrial | 1.82 | 4.72^*^ | 0 |
| LM 16, 24 | Aquatic | Semiaquatic | Terrestrial | LM 17, 23 | Aquatic | Semiaquatic | Terrestrial |
| Aquatic | 0 |  |  | Aquatic | 0 |  |  |
| Semiaquatic | 4.46^**^ | 0 |  | Semiaquatic | 1.13 | 0 |  |
| Terrestrial | 0.12 | 4.34^**^ | 0 | Terrestrial | 5.39 | 1.67 | 0 |
| LM 18, 22 | Aquatic | Semiaquatic | Terrestrial | LM 19, 21 | Aquatic | Semiaquatic | Terrestrial |
| Aquatic | 0 |  |  | Aquatic | 0 |  |  |
| Semiaquatic | 0.31 | 0 |  | Semiaquatic | 1.54 | 0 |  |
| Terrestrial | 0.45 | 0.14 | 0 | Terrestrial | 0.92 | 2.46 | 0 |
| LM 20 | Aquatic | Semiaquatic | Terrestrial |  |  |  |  |
| Aquatic | 0 |  |  |  |  |  |  |
| Semiaquatic | 7.66^**^ | 0 |  |  |  |  |  |
| Terrestrial | 1.01 | 8.66^**^ | 0 |  |  |  |  |

D Pairwise comparison and corresponding *p*-values of single landmark point of the palate calculated as procrustes variances (× E-5) for 34 species grouped by life history strategy. *, *p* <= 0.05.

| LM 1, 14 | Metamorphosis | Neoteny | LM 2, 13 | Metamorphosis | Neoteny | LM 3, 12 | Metamorphosis | Neoteny |
| --- | --- | --- | --- | --- | --- | --- | --- | --- |
| Metamorphosis | 0 |  | Metamorphosis | 0 |  | Metamorphosis | 0 |  |
| Neoteny | 6.55 | 0 | Neoteny | 8.37 | 0 | Neoteny | 4.11 | 0 |
| LM 4, 11 | Metamorphosis | Neoteny | LM 5, 10 | Metamorphosis | Neoteny | LM 6, 9 | Metamorphosis | Neoteny |
| Metamorphosis | 0 |  | Metamorphosis | 0 |  | Metamorphosis | 0 |  |
| Neoteny | 4.54 | 0 | Neoteny | 70.98 | 0 | Neoteny | 65.45 | 0 |
| LM 7, 8 | Metamorphosis | Neoteny | LM 15 | Metamorphosis | Neoteny | LM 16, 24 | Metamorphosis | Neoteny |
| Metamorphosis | 0 |  | Metamorphosis | 0 |  | Metamorphosis | 0 |  |
| Neoteny | 26.36 | 0 | Neoteny | 29.63^*^ | 0 | Neoteny | 13.60 | 0 |
| LM 17, 23 | Metamorphosis | Neoteny | LM 18, 22 | Metamorphosis | Neoteny | LM 19, 21 | Metamorphosis | Neoteny |
| Metamorphosis | 0 |  | Metamorphosis | 0 |  | Metamorphosis | 0 |  |
| Neoteny | 4.90 | 0 | Neoteny | 3.58 | 0 | Neoteny | 9.17 | 0 |
| LM 20 | Metamorphosis | Neoteny |  |  |  |  |  |  |
| Metamorphosis | 0 |  |  |  |  |  |  |  |
| Neoteny | 14.66 | 0 |  |  |  |  |  |  |

E Pairwise comparison and corresponding *p*-values of single landmark point of the palate calculated as procrustes variances (× E-5) for 34 species grouped by taxonomic affiliation. *, *p* <= 0.05; **, *p* <= 0.01.

| LM 1, 14 | basal cryptobranchoids | Cryptobranchidae | Hynobiidae | non-cryptobranchoids | Stem Hynobiidae |
| --- | --- | --- | --- | --- | --- |
| basal cryptobranchoids | 0 |  |  |  |  |
| Cryptobranchidae | 11.17 | 0 |  |  |  |
| Hynobiidae | 9.78 | 20.95 | 0 |  |  |
| non-cryptobranchoids | 22.56 | 33.73 | 12.78 | 0 |  |
| Stem Hynobiidae | 15.20 | 26.36 | 5.41 | 7.36 | 0 |
| LM 2, 13 | basal cryptobranchoids | Cryptobranchidae | Hynobiidae | non-cryptobranchoids | Stem Hynobiidae |
| basal cryptobranchoids | 0 |  |  |  |  |
| Cryptobranchidae | 8.86 | 0 |  |  |  |
| Hynobiidae | 19.98 | 11.12 | 0 |  |  |
| non-cryptobranchoids | 19.84 | 10.98 | 0.14 | 0 |  |
| Stem Hynobiidae | 15.55 | 6.69 | 4.43 | 4.29 | 0 |
| LM 3, 12 | basal cryptobranchoids | Cryptobranchidae | Hynobiidae | non-cryptobranchoids | Stem Hynobiidae |
| basal cryptobranchoids | 0 |  |  |  |  |
| Cryptobranchidae | 13.60 | 0 |  |  |  |
| Hynobiidae | 14.72 | 1.12 | 0 |  |  |
| non-cryptobranchoids | 33.83 | 20.22 | 19.11 | 0 |  |
| Stem Hynobiidae | 9.28 | 4.32 | 5.44 | 24.54 | 0 |
| LM 4, 11 | basal cryptobranchoids | Cryptobranchidae | Hynobiidae | non-cryptobranchoids | Stem Hynobiidae |
| basal cryptobranchoids | 0 |  |  |  |  |
| Cryptobranchidae | 17.86 | 0 |  |  |  |
| Hynobiidae | 2.05 | 15.82 | 0 |  |  |
| non-cryptobranchoids | 23.44 | 41.30^*^ | 25.48 | 0 |  |
| Stem Hynobiidae | 4.13 | 13.73 | 2.09 | 27.57 | 0 |
| LM 5, 10 | basal cryptobranchoids | Cryptobranchidae | Hynobiidae | non-cryptobranchoids | Stem Hynobiidae |
| basal cryptobranchoids | 0 |  |  |  |  |
| Cryptobranchidae | 35.81 | 0 |  |  |  |
| Hynobiidae | 138.27 | 102.46 | 0 |  |  |
| non-cryptobranchoids | 117.45 | 81.64 | 20.82 | 0 |  |
| Stem Hynobiidae | 87.22 | 51.41 | 51.05 | 30.23 | 0 |
| LM 6, 9 | basal cryptobranchoids | Cryptobranchidae | Hynobiidae | non-cryptobranchoids | Stem Hynobiidae |
| basal cryptobranchoids | 0 |  |  |  |  |
| Cryptobranchidae | 27.33 | 0 |  |  |  |
| Hynobiidae | 48.38 | 75.72 | 0 |  |  |
| non-cryptobranchoids | 21.50 | 5.84 | 69.88 | 0 |  |
| Stem Hynobiidae | 41.92 | 69.26 | 6.46 | 63.42 | 0 |
| LM 7, 8 | basal cryptobranchoids | Cryptobranchidae | Hynobiidae | non-cryptobranchoids | Stem Hynobiidae |
| basal cryptobranchoids | 0 |  |  |  |  |
| Cryptobranchidae | 6.17 | 0 |  |  |  |
| Hynobiidae | 6.75 | 12.92 | 0 |  |  |
| non-cryptobranchoids | 102.16^*^ | 108.33^**^ | 95.41^**^ | 0 |  |
| Stem Hynobiidae | 21.75 | 27.92 | 15.01 | 80.40^**^ | 0 |
| LM 15 | basal cryptobranchoids | Cryptobranchidae | Hynobiidae | non-cryptobranchoids | Stem Hynobiidae |
| basal cryptobranchoids | 0 |  |  |  |  |
| Cryptobranchidae | 7.26 | 0 |  |  |  |
| Hynobiidae | 25.25 | 17.99 | 0 |  |  |
| non-cryptobranchoids | 1.58 | 8.84 | 26.83 | 0 |  |
| Stem Hynobiidae | 59.08^*^ | 51.82^*^ | 33.83^*^ | 60.66^*^ | 0 |
| LM 16, 24 | basal cryptobranchoids | Cryptobranchidae | Hynobiidae | non-cryptobranchoids | Stem Hynobiidae |
| basal cryptobranchoids | 0 |  |  |  |  |
| Cryptobranchidae | 0.48 | 0 |  |  |  |
| Hynobiidae | 9.40 | 8.92 | 0 |  |  |
| non-cryptobranchoids | 0.44 | 0.04 | 8.96 | 0 |  |
| Stem Hynobiidae | 33.05^*^ | 32.57^*^ | 23.65^*^ | 32.61^*^ | 0 |
| LM 17, 23 | basal cryptobranchoids | Cryptobranchidae | Hynobiidae | non-cryptobranchoids | Stem Hynobiidae |
| basal cryptobranchoids | 0 |  |  |  |  |
| Cryptobranchidae | 14.04 | 0 |  |  |  |
| Hynobiidae | 13.56 | 0.48 | 0 |  |  |
| non-cryptobranchoids | 11.10 | 2.94 | 2.46 | 0 |  |
| Stem Hynobiidae | 17.55 | 3.51 | 3.99 | 6.45 | 0 |
| LM 18, 22 | basal cryptobranchoids | Cryptobranchidae | Hynobiidae | non-cryptobranchoids | Stem Hynobiidae |
| basal cryptobranchoids | 0 |  |  |  |  |
| Cryptobranchidae | 0.46 | 0 |  |  |  |
| Hynobiidae | 3.34 | 3.80 | 0 |  |  |
| non-cryptobranchoids | 17.01 | 17.47 | 13.67 | 0 |  |
| Stem Hynobiidae | 0.36 | 0.10 | 3.70 | 17.37 | 0 |
| LM 19, 21 | basal cryptobranchoids | Cryptobranchidae | Hynobiidae | non-cryptobranchoids | Stem Hynobiidae |
| basal cryptobranchoids | 0 |  |  |  |  |
| Cryptobranchidae | 50.46 | 0 |  |  |  |
| Hynobiidae | 35.34 | 15.13 | 0 |  |  |
| non-cryptobranchoids | 6.29 | 44.18 | 29.05 | 0 |  |
| Stem Hynobiidae | 9.93 | 40.53 | 25.40 | 3.65 | 0 |
| LM 20 | basal cryptobranchoids | Cryptobranchidae | Hynobiidae | non-cryptobranchoids | Stem Hynobiidae |
| basal cryptobranchoids | 0 |  |  |  |  |
| Cryptobranchidae | 9.44 | 0 |  |  |  |
| Hynobiidae | 1.38 | 8.07 | 0 |  |  |
| non-cryptobranchoids | 56.86 | 66.30^*^ | 58.23^*^ | 0 |  |
| Stem Hynobiidae | 22.01 | 31.46 | 23.39 | 34.84 | 0 |

F Absolute morphological disparity of the palate calculated as procrustes variances for 34 species grouped by ecology, life history strategy and taxonomic affiliations.

| Category | Groups | Palate (×10^-3^) | Vomer (×10^-3^) | Parasphenoid (×10^-6^) |
| --- | --- | --- | --- | --- |
| Ecology | aquatic | 5.75 | 2.00 | 1.74 |
|  | semiaquatic | 10.02 | 3.28 | 3.45 |
|  | terrestrial | 11.47 | 4.71 | 2.05 |
| Life history | neoteny | 6.89 | 2.45 | 1.99 |
|  | metamorphosis | 9.28 | 3.61 | 2.06 |
| Taxonomic affiliations | Hynobiidae | 9.20 | 3.71 | 1.79 |
|  | stem hynobiids | 9.62 | 3.22 | 3.19 |
|  | Pancryptobrancha | 3.57 | 1.30 | 0.96 |
|  | basal Cryptobranchoidea | 4.97 | 1.35 | 2.27 |
|  | non-Cryptobranchoidea | 11.48 | 4.33 | 2.83 |
|  | 34 species | Fig. 3 | Fig. 3 | Fig. 3 |

G Absolute morphological disparity calculated as procrustes variances and evolutionary rates for each landmark point of the palate across 34 species grouped by ecology, life history and taxonomic affiliations.

| Landmarks | 1, 14 | 2, 13 | 3, 12 | 4, 11 | 5, 10 | 6, 9 | 7, 8 | 15 | 16, 24 | 17, 23 | 18, 22 | 19, 21 | 20 |
| --- | --- | --- | --- | --- | --- | --- | --- | --- | --- | --- | --- | --- | --- |
| aquatic (×10^-4^) | 2.57 | 2.83 | 1.44 | 1.60 | 5.79 | 2.63 | 3.21 | 1.15 | 1.28 | 1.66 | 1.75 | 2.24 | 2.37 |
| semiaquatic (×10^-4^) | 10.60 | 3.72 | 0.22 | 4.01 | 2.69 | 11.44 | 1.50 | 7.68 | 5.74 | 5.29 | 1.44 | 0.70 | 10.03 |
| terrestrial (×10^-4^) | 3.05 | 2.58 | 1.46 | 2.53 | 22.08 | 13.25 | 2.17 | 2.96 | 1.40 | 2.20 | 1.30 | 3.17 | 1.36 |
| neoteny (×10^-4^) | 3.31 | 2.66 | 1.79 | 2.46 | 6.93 | 2.68 | 4.64 | 0.38 | 0.69 | 2.14 | 1.7 | 3.3 | 3.84 |
| metamorphosis (×10^-4^) | 3.97 | 3.5 | 1.38 | 2.01 | 14.03 | 9.22 | 2 | 3.35 | 2.05 | 1.65 | 1.34 | 2.38 | 2.38 |
| Hynobiidae (×10^-4^) | 3.86 | 3.58 | 1.48 | 2.05 | 15.01 | 9.34 | 1.72 | 2.7 | 1.6 | 1.73 | 1.41 | 1.89 | 1.93 |
| stem hynobiids (×10^-4^) | 4.4 | 3.14 | 0.94 | 1.84 | 9.91 | 8.7 | 3.22 | 6.08 | 3.97 | 1.33 | 1.04 | 4.43 | 4.26 |
| Pancryptobrancha (×10^-4^) | 1.77 | 2.47 | 1.37 | 0.47 | 4.77 | 1.77 | 0.42 | 0.9 | 0.71 | 1.68 | 1.03 | 0.38 | 1.12 |
| basal Cryptobranchoidea (×10^-4^) | 2.89 | 1.59 | 1.27 | 2.25 | 1.19 | 4.51 | 1.04 | 0.17 | 0.66 | 3.08 | 1.08 | 5.43 | 2.06 |
| non-Cryptobranchoidea (×10^-4^) | 5.14 | 3.57 | 3.4 | 4.6 | 12.93 | 2.36 | 11.26 | 1.32 | 0.7 | 1.97 | 2.78 | 4.8 | 7.75 |
| evolutionary rate (×10^-6^) | 11.37 | 9.26 | 3.28 | 6.63 | 20.98 | 13.97 | 8.42 | 8.85 | 6.62 | 4.04 | 3.95 | 6.53 | 7.43 |

H Comparison of evolutionary rates of the palate and centroid size for 34 species grouped by ecology, life history and taxonomic affiliations. *, *p* <= 0.05; **, *p* <= 0.01; ***, *p* <= 0.001.

| Category | Groups | Palate (×10^-6^) | | Vomer (×10^-6^) | | Parasphenoid (×10^-6^) | log (CS) (×10^-4^) |
| --- | --- | --- | --- | --- | --- | --- | --- |
| Ecology | aquatic | | 8.02^**^ | | 9.26^**^ | 6.29^***^ | 28.53 |
|  | semiaquatic | | 26.02^**^ | | 30.38^**^ | 19.90^***^ | 3.63 |
|  | terrestrial | | 6.93^**^ | | 9.39^**^ | 3.49^***^ | 22.09 |
| Life history | neoteny | | 8.91 | | 10.96 | 6.04 | 27.25 |
|  | metamorphosis | | 8.50 | | 10.44 | 5.80 | 23.29 |
| Taxonomic affiliations | Hynobiidae | | 6.43^*^ | | 8.13 | 4.06^**^ | 20.04 |
|  | stem hynobiids | | 19.42^*^ | | 21.75 | 16.16^**^ | 4.85 |
|  | Pancryptobrancha | | 7.73^*^ | | 9.60 | 5.12^**^ | 36.23 |
|  | basal Cryptobranchoidea | | 8.90^*^ | | 12.39 | 4.00^**^ | 83.51 |
|  | non-Cryptobranchoidea | | 6.40^*^ | | 8.68 | 3.20^**^ | 34.28 |
|  | 34 species | | Fig. 3 | | 10.56^***^ | 5.85^***^ | — |

I Contingency table showing the association between ecological preference and life history strategy and two discrete characters of vomerine tooth row (position and arrangement pattern) across 34 species represented by 70 specimens.

| Ecology and  Life history | Vomerine tooth row position (VTRP) | | | | | | | | |
| --- | --- | --- | --- | --- | --- | --- | --- | --- | --- |
|  | anterior | | mid-posterior | | middle | | posterior | |  |
|  | *n* | % | *n* | % | *n* | % | *n* | % | Total |
| Aquatic | 10 | 100.00 | 8 | 42.11 | 13 | 100.00 | 5 | 17.86 | 36 |
| Semiaquatic | 0 | 0.00 | 3 | 15.79 | 0 | 0 | 1 | 3.57 | 4 |
| Terrestrial | 0 | 0.00 | 8 | 42.11 | 0 | 0 | 22 | 78.57 | 30 |
| Total | 10 | 14.29 | 19 | 27.14 | 13 | 18.57 | 28 | 40.00 | 70 |
| Cochran-Mantel-Haenszel statistics for VTRP ~ ecological preference | | | | | | | | | |
| Statistic alternative hypothesis | | | | DF | | Value | | Probability | |
| 1 Nonzero correlation | | | | 1 | | 16.07 | | < 0.0001 | |
| 2 Row mean scores differ | | | | 2 | | 16.59 | | 0.0003 | |
| 3 General association | | | | 6 | | 39.54 | | < 0.0001 | |
| Metamorphosis | 0 | 0.00 | 19 | 100.00 | 9 | 69.23 | 27 | 96.43 | 55 |
| Neoteny | 10 | 100.00 | 0 | 0.00 | 4 | 30.77 | 1 | 3.57 | 15 |
| Total | 10 | 14.29 | 19 | 27.14 | 13 | 18.57 | 28 | 40.00 | 70 |
| Cochran-Mantel-Haenszel statistics for VTRP ~ life history strategy | | | | | | | | | |
| Statistic alternative hypothesis | | | | DF | | Value | | Probability | |
| 1 Nonzero correlation | | | | 1 | | 19.02 | | < 0.0001 | |
| 2 Row mean scores differ | | | | 1 | | 19.02 | | < 0.0001 | |
| 3 General association | | | | 3 | | 47.14 | | < 0.0001 | |
| Ecology and  Life history | **Vomerine tooth row arrangement (VTRA)** | | | | | | | | |
|  | oblique | | parallel | | | Transverse | | |  |
|  | *n* | % | *n* | % | | *n* | % | | Total |
| Aquatic | 5 | 100.00 | 21 | 100.00 | | 10 | 22.73 | | 36 |
| Semiaquatic | 0 | 0.00 | 0 | 0.00 | | 4 | 9.09 | | 4 |
| Terrestrial | 0 | 0.00 | 0 | 0.00 | | 30 | 68.18 | | 30 |
| Total | 5 | 7.14 | 21 | 30.00 | | 44 | 62.86 | | 70 |
| Metamorphosis | 4 | 80.00 | 7 | 33.33 | | 44 | 100.00 | | 55 |
| Neoteny | 1 | 20.00 | 14 | 66.67 | | 0 | 0.00 | | 15 |
| Total | 5 | 7.14 | 21 | 30.00 | | 44 | 62.86 | | 70 |
| Cochran-Mantel-Haenszel statistics for VTRA ~ life history strategy | | | | | | | | | |
| Statistic alternative hypothesis | | | | DF | | Value | Probability | | |
| 1 Nonzero correlation | | | | 1 | | 18.80 | < 0.0001 | | |
| 2 Row mean scores differ | | | | 1 | | 18.80 | < 0.0001 | | |
| 3 General association | | | | 2 | | 37.00 | < 0.0001 | | |

J Non-shape covariates of the palate of 35 species and the ancestral states for internal nodes reconstructed by the “Rphylopars” R package.

| Taxa/internal node # corresponding to fig. S33b | Parasphenoid/Palate length | Vomer/Palate length | Outer/Inner VTR ratio | VTR/Vomer width | Teeth # on a single vomer |
| --- | --- | --- | --- | --- | --- |
| *Karaurus sharovi* | 0.79 | 0.28 | 2.96 | 0.97 | 14 |
| *Kokartus honorarius* | 0.86 | 0.16 | 2.87 | 0.95 | 9 |
| *Beiyanerpeton jianpingense* | 0.89 | 0.21 | 1.62 | 0.98 | 16 |
| *Chunerpeton tianyiense* | 0.90 | 0.25 | 1.71 | 0.91 | 25 |
| *Pangerpeton sinense* | 0.79 | 0.46 | 0.86 | 0.79 | 30 |
| *Aviturus exsecratus* | 0.77 | 0.39 | 1.08 | 0.59 | 16 |
| *Andrias davidianus* | 0.84 | 0.29 | 3.11 | 0.95 | 17 |
| *Andrias japonicus* | 0.82 | 0.33 | 2.81 | 0.95 | 20 |
| *Cryptobranchus alleganiensis* | 0.83 | 0.32 | 2.34 | 0.99 | 21 |
| *Linglongtriton daxishanensis* | 0.71 | 0.34 | 0.64 | 0.76 | 15 |
| *Neimengtriton daohugouensis* | 0.61 | 0.44 | 0.68 | 0.78 | 16 |
| *Nuominerpeton aquilonare* | 0.79 | 0.34 | 1.16 | 0.57 | 14 |
| *Liaoxitriton zhongjiani* | 0.79 | 0.32 | 1.03 | 0.57 | 11 |
| *Ranodon sibiricus* | 0.81 | 0.29 | 1.20 | 0.46 | 8 |
| *Paradactylodon mustersi* | 0.86 | 0.28 | 1.96 | 0.45 | 8 |
| *Paradactylodon persicus* | 0.77 | 0.35 | 0.82 | 0.56 | 15 |
| *Pachyhynobius shangchengensis* | 0.81 | 0.33 | 0.32 | 0.35 | 10 |
| *Salamandrella keyserlingii* | 0.73 | 0.38 | 0.46 | 0.61 | 23 |
| *Protohynobius puxiongensis* | 0.80 | 0.30 | 0.85 | 0.63 | 12 |
| *Pseudohynobius shuichengensis* | 0.75 | 0.34 | 0.69 | 0.63 | 15 |
| *Pseudohynobius kuankuoshuiensis* | 0.79 | 0.31 | 0.57 | 0.61 | 14 |
| *Pseudohynobius guizhouensis* | 0.74 | 0.33 | 0.56 | 0.65 | 15 |
| *Pseudohynobius jinfo* | 0.76 | 0.32 | 0.84 | 0.61 | 14 |
| *Pseudohynobius flavomaculatus* | 0.74 | 0.33 | 0.70 | 0.63 | 14 |
| *Liua tsinpaensis* | 0.79 | 0.30 | 1.41 | 0.53 | 9 |
| *Liua shihi* | 0.85 | 0.26 | 1.60 | 0.45 | 7 |
| *Batrachuperus yenyuanensis* | 0.84 | 0.29 | 1.88 | 0.36 | 4 |
| *Batrachuperus pinchonii* | 0.82 | 0.27 | 1.51 | 0.41 | 6 |
| *Batrachuperus londongensis* | 0.85 | 0.28 | 1.49 | 0.26 | 4 |
| *Batrachuperus tibetanus* | 0.84 | 0.28 | 1.30 | 0.40 | 6 |
| *Batrachuperus karlschmidti* | 0.85 | 0.28 | 1.53 | 0.33 | 4 |
| *Hynobius nebulosus* | 0.80 | 0.44 | 0.43 | 0.66 | 25 |
| *Hynobius amjiensis* | 0.79 | 0.46 | 0.38 | 0.62 | 31 |
| *Onychodactylus japonicus* | 0.80 | 0.29 | 0.85 | 0.71 | 16 |
| *Regalerpeton weichangense* | 0.91 | 0.16 | 1.65 | 0.95 | 16 |
| 36 (common ancestor of Caudata) | 0.83 | 0.27 | 1.88 | 0.90 | 18 |
| 37 | 0.83 | 0.23 | 2.55 | 0.94 | 14 |
| 38 (common ancestor of Urodela) | 0.83 | 0.28 | 1.70 | 0.89 | 19 |
| 39 (common ancestor of Cryptobranchoidea) | 0.82 | 0.30 | 1.54 | 0.87 | 20 |
| 40 | 0.80 | 0.33 | 1.34 | 0.84 | 20 |
| 41 | 0.78 | 0.33 | 1.25 | 0.82 | 19 |
| 42 (common ancestor of Pancryptobrancha) | 0.79 | 0.35 | 1.57 | 0.78 | 18 |
| 43 | 0.82 | 0.32 | 2.40 | 0.92 | 19 |
| 44 | 0.83 | 0.31 | 2.90 | 0.95 | 19 |
| 45 (common ancestor of Panhynobia) | 0.76 | 0.33 | 1.11 | 0.81 | 17 |
| 46 | 0.71 | 0.36 | 0.90 | 0.78 | 16 |
| 47 | 0.67 | 0.39 | 0.82 | 0.76 | 16 |
| 48 | 0.76 | 0.35 | 1.02 | 0.62 | 13 |
| 49 | 0.88 | 0.20 | 1.50 | 0.90 | 16 |
| 50 (common ancestor of Hynobiidae) | 0.84 | 0.25 | 1.25 | 0.76 | 15 |
| 51 | 0.80 | 0.32 | 0.99 | 0.54 | 14 |
| 52 | 0.81 | 0.31 | 1.14 | 0.52 | 12 |
| 53 | 0.81 | 0.31 | 1.25 | 0.51 | 12 |
| 54 | 0.80 | 0.33 | 0.92 | 0.54 | 14 |
| 55 | 0.80 | 0.33 | 0.85 | 0.53 | 15 |
| 56 | 0.80 | 0.33 | 0.93 | 0.54 | 15 |
| 57 | 0.80 | 0.32 | 1.06 | 0.52 | 12 |
| 58 | 0.80 | 0.32 | 1.06 | 0.53 | 12 |
| 59 | 0.78 | 0.32 | 0.86 | 0.59 | 13 |
| 60 | 0.77 | 0.32 | 0.77 | 0.61 | 14 |
| 61 | 0.77 | 0.33 | 0.70 | 0.62 | 14 |
| 62 | 0.77 | 0.32 | 0.65 | 0.62 | 14 |
| 63 | 0.76 | 0.33 | 0.77 | 0.62 | 14 |
| 64 | 0.81 | 0.29 | 1.37 | 0.50 | 9 |
| 65 | 0.83 | 0.29 | 1.46 | 0.40 | 7 |
| 66 | 0.84 | 0.28 | 1.46 | 0.37 | 6 |
| 67 | 0.84 | 0.28 | 1.48 | 0.35 | 5 |
| 68 | 0.84 | 0.28 | 1.44 | 0.37 | 5 |
| 69 | 0.80 | 0.41 | 0.58 | 0.61 | 24 |

K Definition of the 24 landmarks for the palate (vomer and parasphenoid) in this study.

| **Landmarks** | **Descriptions** |
| --- | --- |
| **LM 1** | The most anterior point of the right vomer |
| **LM 2** | The most posterolateral point of the choanal notch of the right vomer |
| **LM 3** | The most anteromedial point of the choanal notch in the right vomer [in some neotenic taxa, this landmark overlaps with Landmark 2] |
| **LM 4** | The most posteromedial point of the choanal notch of the right vomer |
| **LM 5** | The most posterior point of the right vomer |
| **LM 6** | Intersection of the most posteromedial part of the right vomer with the sagittal axis of the skull [in some taxa, landmarks 6 and 5 can overlap with each other] |
| **LM 7** | Intersection point between the median border of the right vomer with the posterior margin of the anteromedial fenestra [in some taxa, landmarks 7 and 1 can overlap with each other] |
| **LM 8** | Intersection point between the median border of the left vomer with the posterior margin of the anteromedial fenestra [in some taxa, landmarks 8 and 14 can overlap with each other] |
| **LM 9** | Intersection of the most posteromedial part of the left vomer with the sagittal axis |
| **LM 10** | The most posterior point of the left vomer [in some taxa, landmarks 10 and 9 can overlap with each other] |
| **LM 11** | The most posteromedial point of the choanal notch of the left vomer |
| **LM 12** | The most anteromedial point of the choanal notch in the left vomer |
| **LM 13** | The most posterolateral point of the left vomer [in some taxa, landmarks 13 and 12 can overlap with each other] |
| **LM 14** | The most anterior point of the left vomer |
| **LM 15** | The most anterior point of the parasphenoid |
| **LM 16** | The most right anterolateral point of the cultriform process of the parasphenoid |
| **LM 17** | Point where the right orbitosphenoid posteriorly articulates with the parasphenoid or the midpoint of the right edge of the cultriform process of the parasphenoid in the case when ossified orbitosphenoids are absent |
| **LM 18** | Point where the cultriform process merges with the right lateral ala of the parasphenoid |
| **LM 19** | The most lateral point of the right lateral ala process of the parasphenoid |
| **LM 20** | The most posterior point of the parasphenoid along the sagittal axis |
| **LM 21** | The most lateral point of the left ala process of the parasphenoid |
| **LM 22** | Point where the cultriform process articulates with the left lateral ala of the parasphenoid |
| **LM 23** | Point where the left orbitosphenoid posteriorly articulates with the parasphenoid |
| **LM 24** | The most left anterolateral point of the cultriform process of the parasphenoid |
